# Lactate‐induced protein lactylation: A bridge between epigenetics and metabolic reprogramming in cancer

**DOI:** 10.1111/cpr.13478

**Published:** 2023-04-14

**Authors:** Ting Wang, Zeng Ye, Zheng Li, De‐sheng Jing, Gui‐xiong Fan, Meng‐qi Liu, Qi‐feng Zhuo, Shun‐rong Ji, Xian‐jun Yu, Xiao‐wu Xu, Yi Qin

**Affiliations:** ^1^ Department of Pancreatic Surgery Fudan University Shanghai Cancer Center Shanghai China; ^2^ Department of Oncology, Shanghai Medical College Fudan University Shanghai China; ^3^ Shanghai Pancreatic Cancer Institute Shanghai China; ^4^ Pancreatic Cancer Institute Fudan University Shanghai China

## Abstract

Lactate is not only an endpoint of glycolysis but is gradually being discovered to play the role of a universal metabolic fuel for energy via the ‘lactate shuttle’ moving between cells and transmitting signals. The glycolytic‐dependent metabolism found in tumours and fast‐growing cells has made lactate a pivotal player in energy metabolism reprogramming, which enables cells to obtain abundant energy in a short time. Moreover, lactate can provide favourable conditions for tumorigenesis by shaping the acidic tumour microenvironment, recruiting immune cells, etc. and the recently discovered lactate‐induced lactylation moves even further on pro‐tumorigenesis mechanisms of lactate production, circulation and utilization. As with other epigenetic modifications, lactylation can modify histone proteins to alter the spatial configuration of chromatin, affect DNA accessibility and regulate the expression of corresponding genes. What's more, the degree of lactylation is inseparable from the spatialized lactate concentration, which builds a bridge between epigenetics and metabolic reprogramming. Here, we review the important role of lactate in energy reprogramming, summarize the latest finding of lactylation in tumorigenesis and try to explore therapeutic strategies in oncotherapy that can kill two birds with one stone.

## Graphical abstract

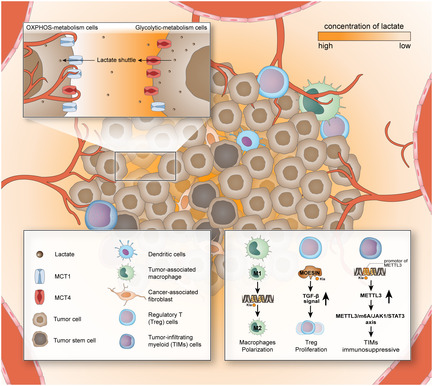

## 1 INTRODUCTION

The correctness of gene expression is controlled not only by DNA sequences but also by epigenetic information. Study shows that a small number of mutations in genes can exhibit profoundly altered DNA methylation patterns.^1^, ^2^ DNA methylation, one of the important epigenetic modifications, is essential for mammalian development, for the deletion of DNA methyltransferases genetically leads to developmental failure.^3^, ^4^ This very first found epigenetic modification 5‐methylcytosine (5mC) later proved to function profoundly in regulating interactions of protein‐DNA, downregulating gene expression, inactivating X chromosome, etc.^5^, ^6^, ^7^, ^8^ Genetic alteration of the epigenome potentially disrupts DNA proper modification patterns without changing its primary base sequence, changes proper nucleosome positioning, induces histone modifications and thus has a profound impact on gene expression.^9^, ^10^

In addition to the disruption of the normal pattern of epigenetic modification such as global hypomethylation of DNA, the occurrence of abnormal epigenetic mutations can also lead to the occurrence of disease.^11^, ^12^, ^13^, ^14^ Epigenetic modification, including DNA modification and histone modification, can lead to gene activation or silence, then, in turn, stirs downstream effects.^15^, ^16^ Therefore, epigenetic changes have long been recognized by researchers as a very important part of disease occurrence, especially in cancer.^17^ Genetic and epigenetic mutation together activated cancer initiation and progression.^10^ Moreover, epigenetic alteration enables tumour cells to escape from chemotherapy and immune surveillance from host.^18^, ^19^ The diverse role of epigenetic change in tumorigenesis and development makes it a promising therapeutic target of cancer.^18^ Drugs that target epigenetic changes include genomic medicines and precision medicines, several of them are put into clinical practice and more have entered clinical trials.^18^, ^20^ Genomic drugs include DNA methyltransferase inhibitors (DNMTi) and histone deacetylase inhibitors (HDACi), which can lead to global changes in the epigenome, while precision medicines target specific genetic alterations in the epigenetic pathways in specific patients.^21^ For example, abnormal promoter DNA hypermethylation‐mediated gene silencing is one of the key characteristics of cancer.^22^ Epigenetic drugs such as DNA methylation inhibitors remove abnormal methylation on the promoter, improving the resulting gene silencing and reactivating abnormal silencing gene expression, in both preclinical and clinical applications have shown good results.^22^, ^23^, ^24^

Epigenetic modifications, including a wide range of post‐translational modifications (PTMs) on histone and non‐histone proteins, integrate environmental cues that feed back into downstream cellular responses through regulatory gene expression.^25^ Histone modification is an important type of epigenetic modification that has been widely detected and exerts different effects at different sites. This covalent modification on histones binds different acyl groups to histone amino acid residues, which, in turn, affects the tightness of histone‐DNA linkages.^26^, ^27^, ^28^ This variation named histone post‐translational modifications (HPTMs) will be amplified during the process of gene expression and determine important biological signals as well as cellular events.^29^ Among these, N‐ε‐lysine acetylation has been widely reported to play a vital role in various cellular processes and thus serves a critical function in tumorigenesis.^25^ Subsequently, lysine acetylation of more histone and non‐histone proteins has been studied and reported intensively. Targeting regulators of lysine acetylation, such as acetyltransferases (writer) and deacetylases (eraser), have been shown to have promising potential to treat human diseases, including cancer.^30^

In 2019, Zhang et al. discovered a brand new epigenetic modification called lactylation on lysine residues that directly stimulates gene transcription from chromatin, through a mass shift from mass spectrometry that matched with a lactyl group.^31^ This new study establishes a novel function for lactate, which utilizes histone lysine lactylation (Kla), regulating gene expression in macrophages.^32^ In macrophages, lactate was reported to induce histone lactylation in the promoters of the profibrotic genes, which is concordant with the upregulation of this very epigenetic modification in the macrophages in fibrotic lungs. Subsequent studies have further shown that lactate, a byproduct of glycolysis, plays the same role that various proteins, lipids and nucleic acids molecules play in mediating cell‐to‐cell regulation in the case of pulmonary fibrosis.^33^ Moreover, the role of histone lactylation in tumorigenesis is gradually found, just as the oncogenic role found in other histone modifications.^34^ Interestingly, lactate itself has long gobbled up the spotlight in the field of metabolic reprogramming. Instead of being considered only as an endpoint and metabolic waste of glycolysis, lactate is now viewed as a universal energy source and signal factor,^35^, ^36^, ^37^, ^38^ shuttling between producer and consumer cells, regulating metabolic pathway, which made it the hub of energy metabolism.^39^, ^40^ As an unimpeded metabolic material in all cells, not to mention its close relationship with energy metabolism, its tumorigenic effect becomes more thought‐provoking.

Collectively, lactate‐induced lactylation, the epigenetic modification that is metabolic stress‐related, links the high‐lactate characteristics of tumour metabolism with tumorigenesis from a new perspective.^39^, ^41^ Given that several studies about lactate‐induced lactylation have emerged, there is still a lack of a thread linking its role in epigenetics and its role in metabolic reprogramming. Here, we combine the classical role of lactate in metabolic reprogramming, with the emerging role of lactate‐induced lactylation in epigenetic inheritance, digging into the close association between the latest found modification lactylation and tumorigenesis, hoping to deepen the integrated understanding of tumour epigenetics and metabolism, laying the foundation for further understanding of its role in disease development and shed some light on new therapeutic strategies.

## 2 METABOLIC REPROGRAMMING DURING CANCER DEVELOPMENT

A major hallmark of cancer is metabolic reprogramming.^42^ This concept originated from Otto Warburg's discovery of an abnormal feature of energy metabolism in cancer cells, known as the Warburg effect, which means even under adequate oxygen conditions, tumour cells still tend to produce lactate through anaerobic respiration for rapid energy production, leading to a state termed ‘aerobic glycolysis’.^43^, ^44^, ^45^, ^46^, ^47^, ^48^, ^49^, ^50^ The causes and mechanisms of strong glycolysis in tumour cells have not been comprehensively elucidated, but it has been proved that cancer cells are induced by oncogenes (such as cMyc) to promote metabolism by enhancing glycolysis, thereby promoting cell proliferation.^51^, ^52^ Pancreatic cancer is a type of tumour with vigorous glycolytic metabolism, and several mutated metabolic genes responsible for such metabolic reprogramming are found. For example, the epigenetic factor protein arginine methyltransferase 5 (PRMT5) silenced the expression of the tumour suppressor F‐box/WD repeat‐containing protein 7 (FBW7) epigenetically, leading to upregulation of cMyc, and subsequently enhanced the aerobic glycolysis in pancreatic cancer cells and its proliferation.^53^ In addition, loss of aerobic glycolysis inhibitors like HIPK is also found related to poor clinical prognosis. Our group found that homeodomain‐interacting protein kinase (HIPK) inhibits the ERK‐cMyc axis, reducing cMyc protein levels and downregulating the expression of the cMyc‐targeted glycolytic gene, thus restraining the proliferation of pancreatic cancer.^54^ More recently, we found that a key enzyme related to the salvage of methionine and adenine, methylthioadenosine phosphorylase (MTAP) is deficient in 20%–30% of pancreatic cancer and metabolic reprogramming mediated by its deletion enhances glycolysis and de novo synthesis of purines in pancreatic cancer cells, resulting in a poor prognosis for pancreatic cancer patients.^55^ These findings reflect the important role of glycolytic upregulation caused by metabolic reprogramming in the development and progression of pancreatic cancer. Moreover, recent studies have further explored and explained the complex metabolic changes and their pattern in the tumour microenvironment and turned the spotlight to lactate, the product of glycolysis. Given that recent studies have shown that lactate rather than pyruvate is the main material in the tricarboxylic acid cycle(TCA), the importance of lactate in energy utilization has been further expanded.^56^

Not only in the tumour microenvironment but also lactate in the human body has long been proven to be not just a product of hypoxia, but can be continuously formed and utilized under aerobic conditions. The production and utilization of lactate not only reflect the metabolism within the cells but also signals through the lactate shuttle between cells.^57^ Glycolysis and the oxidative pathway are not mutually exclusive processes, but interconnected processes, especially through intercellular lactate shuttle to achieve synergy, maximizing the use of substrates to produce energy, and there is increasing evidence of lactate as an important regulator of system metabolic coordination.^58^ And in the tumour microenvironment, this interconnected energy supply is very important.

Some studies have shown that aerobic glycolysis is performed by the cancer‐associated fibroblasts (CAFs) recruited by the tumour, which thus extrudes lactate to ‘feed’ neighbouring cancer cells. The expression of MCT4, a transporter that controls lactate efflux, in CAFs and the high expression of MCT1, a transporter involved in lactate uptake, in breast cancer cells support these ideas. This ‘metabolic coupling’ between the tumour‐associated stroma and adjacent epithelial cancer cells provides the impetus for the growth and metastasis of cancer cells.^59^

However, there is another recent finding that may give a different idea. The hypoxic cell population in the tumour microenvironment uses glucose for Warburg‐like metabolism, and the ensuing large amount of lactate is transported via the lactate shuttle to the adjacent normal oxygen‐supplying cell population for oxidative phosphorylation metabolism (OXPHOS metabolism).^60^ This lactate shuttle facilitates redistribution and efficient utilization of energy substrates, which are also fully used by tumour cells to optimize energy generation and meet their needs of rapid growth via labour division in energy metabolism.^40^, ^60^, ^61^ Interestingly, there exists bidirectional and negative feedback between glycolysis and pH in normal cells, and the lactate shuttle revolving between tumour cells plays an important role in breaking through this feedback limitation to maintain glycolysis flux. A large amount of glycolytic energy supply in a hypoxia microenvironment or aerobic glycolysis‐dependent metabolism of tumour cells can lead to the production of extensive lactate and H+, and the resulting intracellular acidic environment will cause glycolysis inhibition via inhibiting key enzymes of glycolysis.^62^, ^63^ But tumour cells avoid such inhibition by upregulating the monocarboxylate transporter (MCT) and transporting lactate to extracellular, and then further utilize it through the above lactate shuttle.^63^ Even with the upregulation of lactate efflux transporters, elevated lactate levels can be detected in tumour cells dominated by glycolysis, which further supports this view and is testified to be closely related to tumour aggressiveness and poor prognosis.^64^ The efficacy of high‐level lactate in promoting cancer progression has been widely recognized.^65^, ^66^

The above two views seem to indicate that there are different ‘metabolic couplings’ in the complex and diverse tumour microenvironment, and there is still no universal conclusion on who is the bearer of lactate production, but what shapes such metabolic reprogramming have been successively reported in recent years. This tumour‐promoting metabolic switch mediates by transforming growth factor signalling, activating HIF‐1α‐transcription, oncogene activation or tumour suppressor gene loss and maintaining tumour cell survival and development in the harsh tumour microenvironment.^67^

**FIGURE 1:**
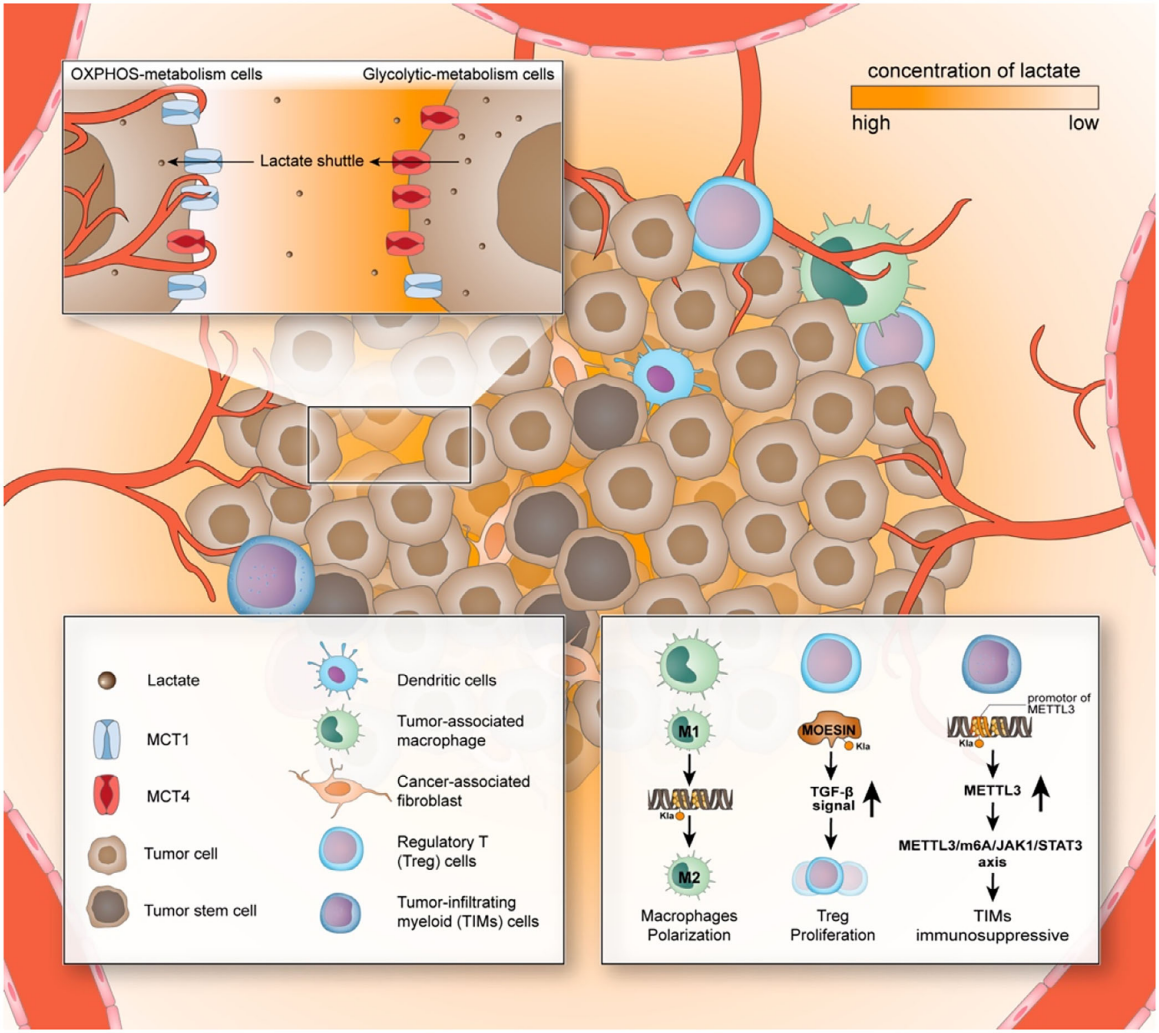
Lactate and lactylation shaped the tumour microenvironment. Tumour cells, tumour‐associated immune cells and tumour‐associated fibroblasts together constitute the tumour microenvironment, and some of them have been found to be regulated by lactate. The most hypoxic part of the tumour microenvironment is often in the state of the highest lactate, and the cells in it are metabolized in a glycolysis‐dependent manner, and the expression of MCT4 is increased that enables transportation of more lactate extracellularly. As the oxygen supply improves, the lactate concentration gradient decreases, and the lactate produced by the ‘lactate‐production cells’ diffuses to adjacent cells along with the concentration gradient. These cells metabolize in an OXPHOS‐dependent manner, and their expression of MCT1 increases, enabling more transformation of ‘borrowed’ lactate into the cell and is used to produce energy in a more efficient way. This shift of energy metabolism reprogramming is also one of the important driving forces for the shaping of the tumour microenvironment, where the high lactate state suppresses the immune cells and recruits cells to push around in various ways, and the formation cause of many pro‐tumorigenesis properties of cells is lactylation. Lactylation can increase the expression of M2‐like genes in macrophages and then urges macrophages to switch from pro‐inflammatory phenotype to anti‐inflammatory phenotype.^31^, ^71^ In Treg cells, lactylation of MOESIN protein can upregulate TGF‐*β* signalling, thereby promoting the proliferation of Treg cells and enhancing their immunosuppressive effect.^76^ In TIM cells, the highly enriched lactylation state at the METTL3 promoter leads to the upregulation of METTL3, which, in turn, exerts their immunosuppressive effect through the METTL3/m6A/JAK1/STAT3 axis.^77^ The significantly increased lactate flux in pancreatic cancer has also been shown to be closely related to the formation of cancer‐associated fibroblasts (CAFs), whether this and lactylation plays a role in the development and progression of pancreatic cancer remains to be explored. Moreover, the acidification of the medium reduces the pH within immune cells and inhibits the activity of a variety of immune cells, including T cells, NK cells and dendritic cells, but whether lactylation has an effect on these immune cells is also a question that remains to be explored.^78^ In addition, many cells closely related to tumour development in the tumour microenvironment have also been found to be able to receive lactate regulation, among which what role lactylation plays remains to be explored, and the exploration of this problem is a promising research direction that may help further revealing the role of epigenetics in shaping the tumour microenvironment.

As mentioned above, in the tumour microenvironment, lactate is mass‐produced, accumulated and promoted tumorigenic through a variety of functions, one of which is reshaping the tumour microenvironment.^68^ As lactate is produced within the cytoplasm, cells excrete it extracellular to ensure intracellular homeostasis and cell survival. The acidic tumour microenvironment, which local high concentrations of lactic acid are responsible for, has been reported to facilitate immunosuppression and promote the invasion and metastasis of tumour cells.^69^, ^70^ High lactate concentrations in tumour biopsies are associated with metastasis and poor clinical outcomes.^60^ The regulation of lactate in the immune microenvironment is also a major means to promote tumour progression. Lactate in the tumour microenvironment can exert the immunosuppressive function, and promote tumour development by inducing, recruiting and regulating immunosuppressive cells.^71^, ^72^ Lactate modifies histones and directly inhibits signalling pathways, thus playing an important role in tumour immunity.^73^, ^74^, ^75^ The discovery of lactate‐induced lactylation indicates that lactate can act on tumorigenesis in an epigenetic modification way in addition to regulating tumorigenesis through the metabolic pathway, filling the gap in the puzzle of the relationship between lactate and cancer with the answer ‘epigenetics’ (Figure 1).

## 3 METABOLITES SERVE AS SUBSTRATES OF EPIGENETIC MODIFICATION

As more and more studies have been reported, many metabolites can become substrates or cofactors of acylating enzymes and act on epigenetic modification. Fluctuations in metabolite concentrations will directly affect the extent of epigenetic modification, this connection can be spatial, which means the transfer of enzymes or metabolites, or changes of them in spatial regions can affect the level of modification in the corresponding region.^79^, ^80^, ^81^, ^82^

Cell chromatin is composed of DNA and histones, both of which can be modified epigenetically, affecting the configuration of chromosomes and, thus, acting as gene regulation. Epigenetic modifications on both DNA and histones regulate the enzymes of the corresponding metabolic pathways, which, in turn, influence epigenetic modification.^83^ DNA methylation directly affects the gene expression of the corresponding sit, and histones have also been proven by many studies to be modified by methylation, phosphorylation, acetylation, lactylation, etc. and affect chromatin packaging and accessibility, thus gene expression.^81^, ^84^ Among the above types of epigenetic modifications, histone lysine acetylation (Kac) is a classic and major histone acylation, which has been extensively studied and proven to play an important role in regulating gene, cell signalling and metabolism and is also intimately related to the occurrence and development of cancer.^25^, ^82^, ^85^

Acetylation is a chemical reaction in which acetyl‐CoA serves as an acetyl‐group donor, lysine acetyltransferases (KATs) and histone acetyltransferases (HATs) as catalytic enzymes, which are also known as writers.^86^ The level of histone acetylation is positively correlated with the level of acetyl‐CoA, and as we have already described, metabolite concentrations closely affect protein modification levels, which also occur in many other metabolite substrate phenomena.^87^, ^88^, ^89^ A variety of HATs have been identified, including three main families, which are p300/CBP, GNAT (Gcn5‐related N‐acetyltransferase) and MYST family.^90^, ^91^, ^92^, ^93^ The removal of acetyl groups (erasers) relies on lysine deacetylase, which balances the activity of HATs, including two families, histone deacetylases (HDACs) and sirtuins,^94^, ^95^ inhibition of which has been found to result in a worse prognosis.^96^ The acetyl group attached to the specific residue of the histone can neutralize the positive charge it carries and increase its hydrophobicity, thereby reducing the affinity of the histone with DNA, loosening the chromatin, making the DNA in it more accessible for transcriptional factor, thus promoting gene expression.^97^, ^98^ Overall, appropriate levels of histone acetylation are important for maintaining normal chromatin structure and mutations in HATs or HDACs have been found in a variety of tumours, with subsequent changes in acetylation levels.^99^, ^100^

Interestingly, recent studies have found that there is a complex crosstalk between the newly discovered histone modification lactylation and acetylation, which may shed some light on their role, separately and synergistically, in tumours and corresponding treatment strategies. Both lactylation and acetylation tend to occur on lysine (Lys) residues, and p300 in the HATs family has also been found to be the main ‘writer’ enzyme for lactylation.^101^, ^102^ Moreover, histone deacetylase (HDACs) 1–3 have also been reported to be the ‘eraser’ of the histone lactylation modification in the site of H3K18la.^103^ Although lactylation has many similarities with acetylation and shares many catalytic enzymes, histone lactylation has a slower kinetic time (24 h) to reach equilibrium than histone acetylation (6 h), indicating that under physiological conditions, the capacity of acetylation is higher than lactylation.^101^ The donor of lactylation was reported to be Lactyl‐CoA.^104^ Since both Lactyl‐CoA and acetyl‐CoA are converted from pyruvate, the production advantages of the two paths oscillate between different cells, producing lactate when the cells lack oxygen supply and sufficient oxygen drives cells into the TCA cycle by converting pyruvate to acetyl‐CoA.^105^ Studies have found that many genes lacking acetylation are present with lactylation.^31^ A competitive relationship between histone lactylation and acetylation arose, and the competitive result, that is, the ratio of lactylation to acetylation can reflect the path of pyruvate transformation, which may also reflect the possibility of whether the cell is prone to malignancy.^101^

## 4 LACTATE‐INDUCED LACTYLATION FUNCTIONS IN TUMOURS VIA REGULATING GENE EXPRESSION METABOLICALLY

### 4.1 Lactylation modification on different substrates

Lactylation was initially found at lysine in histones, which is also known as Kla. After the histone lactylation was discovered, Zhang et al. demonstrated through further research that the lactylation modification also increased in a dose‐dependent manner with the addition of glucose dose, which was further demonstrated by tracking glucose 13C‐labelled atoms. Drug‐mediated inhibition and promotion of glycolysis, respectively, lead to a decrease and increase in global Kla amounts, which means that lactylation levels are inextricably linked to local lactate concentrations.^31^ Stimuli such as hypoxia and microbial exposure can cause cellular energy metabolism to shift to the glycolysis‐dependent pattern, thereby increasing lactate production, increasing local lactate concentrations and stimulating histone Kla.^71^, ^73^ Strahl et al. have proposed that some highly conserved modifications in the histone tail can be combined to form a ‘histone code’ that can be read and excited downstream events, which has since been demonstrated by other studies.^84^, ^106^, ^107^, ^108^ Interestingly, whether lactylation of histones plays a role in this code remains to be further explored.

Kla has been found to play a regulatory role in physiological processes such as activating transcription, promoting cell reprogramming, promoting pulmonary fibrosis and promoting macrophage polarization.^33^, ^109^, ^110^, ^111^ In addition, the role of lactylation has been found in many diseases and pathological processes, and its importance is increasingly recognized by researchers. We summarize recent findings about lactylation in various diseases to analyse modification sites and functions of lactylation (Table 1). So far, more and more lactylation sites in different diseases have been excavated, and some of them play an important role in the development of diseases due to its influence on expression of important functional genes. However, the research on writers and erasers, which are crucial for the occurrence and removal of lactylation, is still in its infancy and remains to be explored. As mentioned above, partially acetylated catalytic enzymes have been found to simultaneously catalyse the occurrence and removal of lactylation and verify their important role in cancer cell genesis and metastasis, such as SIRT2 (Class III HDACs) has been found to act as a histone delactylase mediated the removal of the lactyl group, thereby inhibiting the proliferation and metastasis of neuroblastoma cells.^121^, ^122^ This not only shows the tumour‐promoting effect of lactylation but also provides new ideas for the treatment of targeted lactylation and has broad clinical prospects.

**TABLE 1:** Lactylation sites and corresponding functions and mechanisms, and metabolic enzymes in different diseases.

Diseases	Lactylation sites	Function and mechanisms	Lactyltransferase	References
Lung myofibroblast	Not mentioned	Histone lactylation of macrophage profibrotic gene promoters promotes the expression of profibrotic mediators in macrophages	p300	33
Alzheimer's disease (AD)	H4K12	The positive feedback loop of H4K12 lactylation/PKM2 in microglia drives the pathogenesis of Alzheimer's disease (AD)	Not mentioned	112, 113
Atherosclerosis	Not mentioned	Histone lactylation promotes the expression of the M2 gene, accompanied by the silencing of the M1 gene	Not mentioned	114, 115, 116
Cerebral ischemia–reperfusion injury (CIRI)	Scl25a4, Src25a5	The Ca^2+^ signalling pathway regulates neuronal apoptosis through lactylation	Not mentioned	117
Ulcerative colitis	H3K18	Lactate promotes histone H3K9 acetylation and histone H3K18 lactylation to change the polarization state of macrophages	Not mentioned	118
Insulin resistance	Lysine in HSkMC	The presence of lysine lactylation (Kla) in human skeletal muscle, which is associated with insulin resistance	Not mentioned	119
Polymicrobial sepsis	HMGB1	The lactylated or acetylated HMGB1 released from macrophages increases endothelium permeability via exosome secretion and aggravated sepsis	p300/CBP	120

With the deepening of its research, as well as the breakthrough of lactated protein analysis technology,^123^ the site of lactylation on histones has been increasingly reported, and its modification function on nonhistone proteins has also been discovered and valued gradually.^124^ Studies have found that comprehensive lactylation exists in glycolytic enzymes, and lactylation is conserved in ALDOA (fructose‐bisphosphate Aldolase A), which also means that there is a feedback loop in glycolysis, which is dependent on lactylation.^125^ In addition, studies have reported that lactylation can occur in non‐histone DNA‐binding proteins, macrophages can take up extracellular lactate to promote HMGB1 lactylation, lactylated or acetylated HMGB1 is released outside macrophages through exosomes, activating inflammation that aggravates sepsis, increasing its severity.^120^

### 4.2 The role of lactylation in cancer and the latest findings

Lactate‐induced lactylation modulates and creates a favourable tumour microenvironment and promotes the survival and progression of tumour.^60^, ^78^, ^126^ In addition to the increased lactate concentration that directly shapes the acidic microenvironment and promotes tumour progression and metastasis, lactate‐induced lactylation can also recruit and manage CAFs, tumour‐infiltrating myeloid cells (TIMs; including macrophages, dendritic cells and regulatory T cells) and cancer stem cells (CSCs) in the microenvironment, to achieve the purpose of reshaping the tumour microenvironment and promoting tumour development.^127^

Lactate itself has long been found to promote tumour proliferation by inducing VEGF expression and TAM polarization to an M2‐like phenotype. Tumour‐derived lactate signalling activates macrophages via HIF1α to reach a tumour‐promoting state characterized by upregulated expression of Arg 1 and VEGF.^71^ VEGF in macrophages induces the formation of the new blood vessel, and Arg 1 provides a substrate for cancer cell proliferation to support tumour growth.^128^, ^129^ Studies also reported that in prostate cancer, it was found that lactate can stabilize HIF1α through HIF1α lactylation under normal oxygen conditions and then regulate downstream pathways which further proved the diversified mechanism of lactate and lactylation in tumorigenesis.^130^ In addition, lactate can also promote inflammation development and blood vessel formation in a HIF1α‐independent manner. Similar to HIF1α, NDRG family member 3 (NDRG3) is degraded in a PHD2/VHL‐dependent manner under normoxia conditions. However, under long‐term hypoxic conditions, NDRG3 is protected from degradation by binding to lactate, which, in turn, leads to NDRG3 elevation, activates the RAF–ERK signalling pathway, and controls hypoxia‐related pathophysiological responses, including inflammation and angiogenesis.^131^ This process is exacerbated by lactate‐induced histone lactylation, and the B‐cell adapter for PI3K (BCAP) promotes the reparative transformation of macrophages through histone lactylation.^102^, ^110^ This lactylation‐mediated transition of macrophages from M1‐like polarization to M2‐like polarization, which changes macrophages from pro‐inflammatory to anti‐inflammatory, has also recently been found in atherosclerosis (AS), suggesting that lactylation‐mediated macrophage polarization plays an important role in chronic inflammatory diseases, in addition to tumours.^114^, ^115^, ^116^

TIMs play an important role in tumour immune escape, and lactylation enriched in the promoter of methyltransferase‐like 3 (METTL3) region drives the high expression of METTL3, which, in turn, strengthens the immunosuppressive capacity of TIMs through the METTL3/m6A/JAK1/STAT3 axis.^77^ Regulatory T (Treg) cells exert immunosuppressive effects, normally essential to maintaining immune tolerance. The deficiency of Treg cells can lead to chronic inflammation and even severe autoimmune diseases.^132^ However, Treg cells also play a critical role in maintaining the immunosuppressive state in the tumour microenvironment. Lactylation of MOESIN (membrane‐organizing extension spike protein) at Lys72 modulates the generation of Treg cells by enhancing TGF‐*β* signalling in Treg cells, and patients with hepatocellular carcinoma with a low degree of MOESIN lactylation in Treg cells were more sensitively responding to anti‐PD‐1 treatment. Combination therapy with anti‐PD‐1 and lactate dehydrogenase inhibitors has a stronger anti‐tumour effect than anti‐PD‐1 alone, which also indicates that lactylation has great potential as a target for combination therapy.^76^ Upregulation of lactylation can be detected in fibrotic lungs, and mechanistically, lactate induces histone lactylation of macrophage profibrotic gene promoters, thus promoting fibrosis.^33^ Pancreatic cancer is a tumour closely related to the fibrotic matrix, and studies have shown that the tumour‐mediated lactate flux in pancreatic cancer is closely related to the formation of CAFs, but whether lactylation plays a role in this still remains further study.^133^

In addition to playing an important role in remodelling the microenvironment, lactylation has also been found to play an important role in the regulation of the pluripotency of cells. Gli‐like transcriptional factor 1 (Glis1) binds to promotors of somatic genes and glycolytic genes in the early stage of reprogramming and triggers the epigenome–metabolome–epigenome cascade, which further facilitates cellular reprogramming from senescent cells into pluripotent cells.^111^

Lactylation can work with other epigenetic modifications to co‐act in tumorigenesis. In ocular melanoma, lactylation signals were also found to be significantly enriched in the YTHDF2 (YTH N6‐methyladenosine RNA binding protein 2) promoter region and YTHDF2, one of the m6A code readers, has been reported to function as an oncogene in a variety of tumours.^134^, ^135^ Elevated levels of lactylation in tumour tissue can lead to a poor prognosis for ocular melanoma by facilitating YTHDF2 expression, which drives oncogenesis.^34^

By directly regulating gene expression, lactate‐induced lactylation can act on key tumorigenesis pathways, thereby promoting tumorigenesis and metastasis. Lactylation was found upregulated in clear cell renal cell carcinoma (ccRCC), triggered by inactive von Hippel–Lindau (VHL), which is widely recognized as an essential part of ccRCC formation.^136^ Histone lactylation in ccRCC promotes tumour progression by activating PDGFR*β* (platelet‐derived growth factor receptor *β*) signalling, which, in turn, facilitates histone lactylation, thus forming a positive feedback loop of pro‐tumorigenesis in ccRCC.^137^ In colorectal cancer (CRC), enterobacterial LPS‐inducible LINC00152 can promote tumour invasion and metastasis, and the approach LPS upregulates LINC00152 is introducing histone lactylation on its promoter to reduce the binding efficiency of inhibitor YY1.^138^ In addition, lactylation has also been found to play an important role in tumours such as hepatocellular carcinoma (HCC) and non‐small cell lung cancer (NSCLC), which shows the close relationship between lactylation and tumorigenesis and metastasis(Table 2).

**TABLE 2:** Lactylation sites and corresponding functions and mechanisms, and metabolic enzymes in different cancer types.

Cancer types	Lactylation sites	Lactyltransferase (writers)	Delactylase (erasers)	Function and mechanisms in cancer	Therapeutic clues	References
Colon cancer	H3K18	p300	HDAC1‐3	Increase Mettl3 expression in tumour‐infiltrating myeloid cells via enriched lactylation in the promotor region of Mettl3, promote tumour immune escape by regulating TIMs	p300 inhibitor C646	31, 77
Ocular melanoma	H3K18, global lactylation	EP300	Not mentioned	Activate the expression of oncogene YTHDF2, which identifies m^6^A‐modified PER1 and TP53 mRNAs and promotes their degradation, thus facilitate tumorigenesis	glycolysis inhibitors	34
Clear cell renal cell carcinoma (ccRCC)	H3K18, global lactylation	EP300	Not mentioned	Inactive von Hippel–Lindau (VHL) triggers histone lactylation, which activates PDGFR*β*‐histone lactylation positive feedback loop in ccRCC and facilitate tumorigenesis	glycolysis inhibitors	137
Non‐small cell lung cancer (NSCLC)	H4K8	Not mentioned	Not mentioned	Histone lactylation increases in HK‐1 and IDH 3G promoters, downregulated mRNA levels of glycolytic enzymes (HK‐1, PKM) and upregulated TCA cycle enzymes (SDHA, IDH3G)	Not mentioned	139
Hepatocellular carcinoma (HCC)	Lys72 of MOESIN	Not mentioned	Not mentioned	Lactylation of MOESIN enhances TGF‐*β* signalling in Treg cells via TGF‐*β* receptor I, Upregulate Treg cells and maintaining an immunosuppressive microenvironment	Glycolysis inhibitors	76
	H3K9, H3K56	Not mentioned	Not mentioned	Increased lactylation of H3 histone effectively promotes the progression of hepatocellular carcinoma (HCC) by promoting LCSC proliferation	Demethylzeylasteral (DML)	125
Prostate cancer	HIF1α	Not mentioned	Not mentioned	Stabilizes HIF1α via HIF1α lactylation under normoxia, thus promotes the expression of KIAA1199, which enhances angiogenesis and vasculogenic mimicry (VM)	Not mentioned	130

## 5 CONCLUSION AND PERSPECTIVE

Lactate is a core energy metabolism substrate that plays the role of a key molecule for metabolism between cells in the tumour microenvironment. Some tumour cells and CAFs undergoing metabolic reprogramming can quickly obtain a large amount of energy by aerobic glycolysis and transport the lactate produced in large quantities to other cells in the microenvironment through ‘lactate shuttle’, efficiently supplying substrates for energy‐producing. As a result, the concentration of lactate in the microenvironment and related cells significantly increases, which not only directly shapes the tumour‐promoting acidic microenvironment but also provides a substrate for cellular lactylation in the tumour‐related microenvironment. Studies have shown that the occurrence of lactylation is related to spatial lactate concentration, and multiple key lactylation sites have been detected in the tumour microenvironment, which can recruit tumour‐related immune cells, reshape the microenvironment and cooperate with other epigenetic modifications to promote tumorigenesis, progression, and even directly act on the gene expression of tumour‐related key pathways. Collectively, In the tumour microenvironment, the production, circulation and utilization of lactate have become the ‘right hand’ of tumours in both energy metabolism and epigenetic modification, which plays a role in facilitating tumour onset and progression. Therefore, whether targeted lactylation can be used as an effective means of cancer treatment, or whether targeted lactate production can kill two birds with one stone, while improving the tumour microenvironment, preventing and improving lactylation modification to combat tumours, the exploration of these problems is promisingly moving towards potentially powerful tumour treatment strategies.

## AUTHOR CONTRIBUTIONS

**Yi Qin** conceived of the presented idea; **Ting Wang**, **Zeng Ye**, **Zheng Li**, **De‐sheng Jing** and **Gui‐xiong Fan** wrote the manuscript and prepared the figure and tables; **Meng‐qi Liu**, **Qi‐feng Zhuo** and **Shun‐rong Ji** searched the literature and collected the references; **Yi Qin**, **Xiao‐wu Xu** and **Xian‐jun Yu** supervised the project and critically reviewed and edited the manuscript. All authors read and approved the final manuscript.

## FUNDING INFORMATION

This work was supported by grants from Shanghai Municipal Science and Technology Commission (20ZR1471100), National Natural Science Foundation of China (No. 82141129, 82173281, 82172948, 81972250, 82172577 and U21A20374), Shanghai Municipal Science and Technology Major Project (21JC1401500), Scientific Innovation Project of Shanghai Education Committee (2019‐01‐07‐00‐07‐E00057), Clinical Research Plan of Shanghai Hospital Development Center (SHDC2020CR1006A) and Xuhui District Artificial Intelligence Medical Hospital Cooperation Project (2021–011).

## CONFLICT OF INTEREST STATEMENT

The authors declare that they have no conflict interests.

## Data Availability

Data sharing is not applicable to this article as no new data were created or analyzed in this study.
